# Influence of Gender, Body Mass Index, and Age on the Pharmacokinetics of Itraconazole in Healthy Subjects: Non-Compartmental Versus Compartmental Analysis

**DOI:** 10.3389/fphar.2022.796336

**Published:** 2022-06-15

**Authors:** Milijana N. Miljković, Nemanja Rančić, Aleksandra Kovačević, Bojana Cikota-Aleksić, Ivan Skadrić, Vesna Jaćević, Momir Mikov, Viktorija Dragojević-Simić

**Affiliations:** ^1^ Centre for Clinical Pharmacology, Military Medical Academy, Belgrade, Serbia; ^2^ Medical Faculty of the Military Medical Academy, University of Defence in Belgrade, Belgrade, Serbia; ^3^ Institute of Microbiology and Immunology, University of Belgrade, Faculty of Medicine, Belgrade, Serbia; ^4^ Department for Experimental Toxicology and Pharmacology, National Poison Control Centre, Belgrade, Serbia; ^5^ Department for Chemistry, Faculty of Science, University of Hradec Kralove, Hradec Kralove, Czechia; ^6^ Institute for Pharmacology, Clinical Pharmacology and Toxicology, Faculty of Medicine, University of Novi Sad, Novi Sad, Serbia

**Keywords:** itraconazole, hydroxy-itraconazole, gender, non-compartmental analysis, compartmental analysis, genetic polymorphism, body mass index, age

## Abstract

Itraconazole is a triazole antifungal agent with highly variable pharmacokinetics, with not yet fully identified factors as the source of this variability. Our study aimed to examine the influence of body mass index, gender, and age on the first dose pharmacokinetics of itraconazole in healthy subjects, using pharmacokinetic modeling, non-compartmental *versus* compartmental ones. A total of 114 itraconazole and hydroxy-itraconazole sets of plasma concentrations of healthy subjects of both genders, determined using a validated liquid chromatographic method with mass spectrometric detection (LC-MS), were obtained for pharmacokinetic analyses performed by the computer program Kinetica 5^®^. Genetic polymorphism in *CYP3A4*, *CYP3A5*, *CYP1A1*, *CYP2C9*, and *CYP2C19* was analyzed using PCR-based methods. Multiple linear regression analysis indicated that gender had a significant effect on AUC as the most important pharmacokinetics endpoint, whereas body mass index and age did not show such an influence. Therefore, further analysis considered gender and indicated that both geometric mean values of itraconazole and hydroxy-itraconazole plasma concentrations in men were prominently higher than those in women. A significant reduction of the geometric mean values of C_max_ and AUC and increment of V_d_ in females compared with males were obtained. Analyzed genotypes and gender differences in drug pharmacokinetics could not be related. Non-compartmental and one-compartmental models complemented each other, whereas the application of the two-compartmental model showed a significant correlation with the analysis of one compartment. They indicated a significant influence of gender on itraconazole pharmacokinetics after administration of the single oral dose of the drug, given under fed conditions. Women were less exposed to itraconazole and hydroxy-itraconazole than men due to poorer absorption of itraconazole, its more intense pre-systemic metabolism, and higher distribution of both drug and its metabolite.

## Introduction

Itraconazole was the first orally bioavailable triazole approved by the Food and Drug Administration since 1992 (Maertens, 2004). It is active against numerous dermatophytes and yeasts and a reasonable choice for cutaneous and extracutaneous sporotrichosis, tinea corporis, extensive tinea versicolor, and oropharyngeal candidiasis, as well as systemic use in onychomycosis (Rogers and Krysan, 2018). It is the drug of choice for patients with indolent, nonmeningeal infections due to *B. dermatitidis*, *H. capsulatum*, *P. brasiliensis*, and *Coccidioides immitis*. It is also used in the treatment of invasive aspergillosis in immunocompromised non-neutropenic patients, particularly those with bronchopulmonary and sinuses infection, as well as bronchopulmonary allergic aspergillosis lasting up to 1 year (Walsh et al., 2008). On the contrary, in neutropenic patients with invasive aspergillosis, after hospital treatment with voriconazole, prolonged ambulatory use of itraconazole is recommended (Tissot et al., 2017). Itraconazole, like other azoles, binds to fungal cytochrome P-450 (CYP450) isoenzymes leading to the inhibition of ergosterol synthesis and disrupting fungal membrane-bound enzyme function and its permeability (Lestner and Hope, 2013; De Doncker et al., 2017; Rogers and Krysan, 2018). The highly variable pharmacokinetics of itraconazole can be largely attributed to unpredictable oral bioavailability (Hardin et al., 1988; Allegra et al., 2017). Itraconazole is a weak base [pKa = 3.7] with very high lipophilicity, so water solubility is the rate-limiting step in its absorption from the gastrointestinal tract (GIT) (Abuhelwa et al., 2015; Abuhelwa et al., 2016). This is especially related to capsules, as an oral formulation, particularly in patients having low gastric acidity. Therefore, giving the capsules together with food and acidic drinks increases the oral bioavailability of itraconazole (Yun et al., 2006; Bae et al., 2011). For example, it was shown that the absolute bioavailability, about 55%, is maximal when capsules are taken immediately after a full meal. The plasma protein binding of itraconazole is as high as 99.8%, but due to high lipophilicity, its volume of distribution (V_d_) is very large (more than 700 L) (Heykants et al., 1982; Prentice and Glasmacher, 2005; McEvoy, 2016). Itraconazole undergoes excessive hepatic metabolism, involving the enzyme CYP3A4, into more than 30 metabolites, but it is considered that the main circulating metabolite in human plasma is hydroxy-itraconazole (Heykants et al., 1982; Prentice and Glasmacher, 2005; Bellmann and Smuszkiewicz, 2017). Itraconazole and hydroxy-itraconazole act as CYP3A4 substrates and inhibitors, affecting their own metabolic clearance and those of the other drugs (Garcia et al., 2018). The elimination of itraconazole is biphasic, with a terminal half-life of approximately 20–24 h after a single dose, while the half-life of hydroxy-itraconazole is about 12 h (McEvoy, 2016; Thummel et al., 2018; Datapharm, 2021). Itraconazole is excreted *via* inactive metabolites up to 35% in urine and 54% in feces, while fecal excretion of the unchanged drug varies between 3% and 18% of the given dose (McEvoy, 2016; Bellmann and Smuszkiewicz, 2017). The peak serum level of itraconazole of approximately 150–250 μg/L was detected after a 100 mg single oral dose, while when given daily for 4 weeks to healthy volunteers, values of 621 ± 337 μg/L were reached, suggesting, together with other clinical data, its nonlinear pharmacokinetics (McEvoy, 2016; Thummel et al., 2018; Datapharm, 2021). In accordance with that, mean and standard deviations for trough concentrations were 193 ± 170 μg/L, indicating wide variations between patients. Therefore, therapeutic drug monitoring (TDM) would timely and appropriately guide dose modifications (Allegra et al., 2017; John et al., 2019). As a result of its variable pharmacokinetics, itraconazole is known as a highly variable drug (HVD), meaning that its within-subject variability for PK parameters, the maximum plasma concentration of drug (C_max_), and area under the concentration-time curve (AUC), is larger than 30% (European Medicines Agency, 2010; Dragojević-Simić et al., 2018; Endrenyi and Tothfalusi, 2019). However, sources of its pharmacokinetics variability are not yet fully elucidated, and age, body mass index, ethnicity, sex, and different pharmaceutical formulations are probably involved (Fagiolino et al., 2007; Allegra et al., 2017). It was shown that the elderly display several metabolic and pharmacokinetic changes that could affect the pharmacokinetics of drugs (Shi and Klotz, 2011), but so far, no studies have been performed to confirm these data with itraconazole. Previous studies of itraconazole pharmacokinetics have shown that age affects its pharmacokinetic parameters, especially in children (Bury et al., 2021). On the contrary, no association was found between age and the total body exposures for the itraconazole and hydroxy-itraconazole in the study involving infants, children, and adolescents (Abdel-Rahman et al., 2007). In contrast, the body weight was identified as an important covariate for the population pharmacokinetic three-compartment model of itraconazole in the previously mentioned population (Abdel-Rahman et al., 2007). In order to develop a population pharmacokinetic model of itraconazole and hydroxy-itraconazole for two oral formulations of itraconazole in healthy subjects and perform covariate model building, Abuhelwa et al. (2015) included body weight and sex among other available covariates. They showed that weight affected itraconazole pharmacokinetics, but gender did not. However, Fagiolino et al. indicated the influence of gender on highly variable drugs, such as itraconazole, which showed significant influence on bioequivalence study (Fagiolino et al., 2007). Moreover, to evaluate the pharmacokinetics of orally given itraconazole for antifungal prophylaxis in children, Allegra et al. performed linear regression analysis and showed that gender (*p* = 0.038) is a positive predictor of trough levels, highlighting the need for TDM in this population (Allegra et al., 2017).

Numerous factors related to gender affect the absorption of the drug, its volume of distribution (V_d_), biotransformation of drugs, and/or clearance (Cl) (Heykants et al., 1987; Anderson, 2005; Soldin and Mattison, 2009). These differences are expected to be even more pronounced when hypervariable drugs, such as itraconazole, are considered. Itraconazole pharmacokinetics has been evaluated in many studies after oral administration (Heykants et al., 1987; Yun et al., 2006; Lindsay et al., 2017; Garcia et al., 2018; Thummel et al., 2018; Nakamura et al., 2019). The non-compartmental method has often been used, although it follows multicompartment kinetics.

Therefore, the study aimed to investigate the influence of body mass index, gender, and age on the first dose pharmacokinetics of itraconazole in healthy subjects using pharmacokinetic modeling, non-compartmental *versus* compartmental ones. Detailed consideration of their possible influence on pharmacokinetics is important due to the possible need for dose adjustment enabling the safe and efficient treatment, especially when it concerns hypervariable drugs, such as itraconazole.

## Materials and Methods

### Study Drug

We used itraconazole capsules, hard, equivalent to 100 mg of itraconazole, manufactured by JanssenCilag S.P.A, Latina, Italy (Sporanox^®^) and Slaviamed d. o.o., Belgrade, Serbia (Kanazol^®^). The bioequivalence of the two products has been proven (Dragojević-Simić et al., 2018).

### Study Protocol

The clinical study documents were approved by an Independent Ethics Committee of the hospital (Ethics Committee of the Military Medical Academy, Belgrade), decision number 53/2019, issued on 4 July 2019). The study was strictly conducted in accordance with the principles of ICH GCP and the latest version of the Helsinki Declaration. It is an academic study based on a bioequivalence study on two oral formulations of itraconazole previously approved, performed, and published (Dragojević-Simić et al., 2018).

### Subjects

Thirty-eight healthy volunteers with an average age of 38.0 ± 6.7 years and an average body weight of 78.47 ± 11.86 kg (body mass index between 19 and 30 kg/m^2^) were included in the study. Subjects were informed about the nature, aims of the study, and potential risks. Informed written consent was obtained before any procedure was performed. All the procedures involving healthy volunteers were already described (Dragojević-Simić et al., 2018). Briefly, the selection was based on their medical and medication history, physical examination (body weight, body height, arterial blood pressure, pulse rate, and electrocardiogram), and clinical laboratory evaluation (hematology, biochemistry, urinalysis, human immunodeficiency virus and hepatitis C antibodies, hepatitis B surface antigen, and serum pregnancy test, female only). Any subject of clinically relevant abnormalities, including gastrointestinal disease and malabsorption, was not included in the study (Dragojević-Simić et al., 2018).

### Study Design

Plasma samples for the analytic and pharmacokinetic data were collected from a randomized three-sequence, three-period, two-treatment, partially replicated crossover bioequivalence study comparing reference and test formulations of itraconazole (Dragojević-Simić et al., 2018). Healthy volunteers who participated in a bioequivalence study took postprandially (30 min after serving a standardized high-fat and high-calorie meal) a single capsule (100 mg), separated by a 14-day washout period, three times (European Medicines Agency, 2010; Dragojević-Simić et al., 2018). Overall, there were 38 subjects, with 16 blood samples per subject per one period, 3 treatment periods, and 114 sets of itraconazole and hydroxy-itraconazole plasma concentrations (Dragojević-Simić et al., 2018). Obtained plasma concentrations of itraconazole and hydroxy-itraconazole were used to conduct pharmacokinetic analyses. Genotyping was performed using the DNA extracted from buccal swab samples obtained from 28 healthy volunteers who consented to this analysis.

### Sample Collection and Analytical Method

As already described, venous blood samples were obtained pre-dose (time 0.0) and at the following time points after administration of the drug: 1.0; 2.0, 3.0, 3.5, 4.0, 4.5, 5.0, 5.5, 6.0, 7.0, 9.0, 12.0, 24.0, 36.0, and 72.0 h (Dragojević-Simić et al., 2018). Plasma concentrations of itraconazole and hydroxy-itraconazole were determined using a validated liquid chromatographic method with mass spectrometric detection (LC-MS), as previously described (Patni et al., 2010; European Medicines Agency, 2011; Dragojević-Simić et al., 2018). A detailed description is given as a supplementary file.

### Pharmacokinetic Analysis

Pharmacokinetic parameters using model-independent method were calculated due to the concentration-time data for itraconazole and hydroxy-itraconazole in plasma (Kinetica software version 5.0). Pharmacokinetics variables from the non-compartmental analysis were peak concentration C_max_, estimated from the maximum observed concentration for itraconazole and hydroxy-itraconazole; time to reach peak concentration t_max_; area under the concentration-time curve AUC_72h_, calculated by the linear trapezoidal rule from time 0 to the time of the last sample with the quantifiable concentration (C_72_); and area under the concentration-time curve from time 0 extrapolated to infinity AUC_∞_, calculated as AUC_72h_ + Ct/k_e_. As the gender differences in pharmacokinetics should be examined and anthropometric differences between men and women relevant for this topic were considered (Abuhelwa et al., 2016), the values of the C_max_, AUC_72h_, and AUC_∞_ parameters were corrected according to the body weight of the subjects. In other words, their value was divided by the ratio of the given dose of the drug (100 mg) and the body weight expressed in kilograms (expressed as C_maxcorr_, AUC_72hcorr_, and AUC_∞ corr_, respectively). The terminal elimination rate constant was determined by least-squares regression analysis during the terminal log-linear phase of the concentration-time curve k_e_, and terminal half-life t_1/2_ was calculated as ln2/k_e_ for itraconazole and hydroxy-itraconazole.

### Pharmacokinetic Variables and Compartmental Analysis

The absorption rate constant k_a_ describes the rate at which a drug enters the system, expressed in units of time −1. *k*_*a*_ is related to the absorption half-life (t1/2a) according to the following equation: ka = ln (2)/t1/2ka. Lag time is denoted as t_lag_ and the calculated maximum concentration as C_maxcalc_; t_maxcalc_ is the time where t = C_max_. The area under the concentration-time curve is denoted as AUC. C_maxcalc_ and AUC were corrected according to the body weight of the subjects. Their value was divided by the ratio of the given dose of the drug (100 mg) and the body weight expressed in kilograms (C_maxcalc corr_ and AUC_corr_). The oral apparent volume of the central or plasma compartment in a two-compartment model is V1/F. The apparent volume of distribution after oral administration in the one-compartment model is V_d_/F. The apparent volume of distribution during the terminal phase after oral administration is V_z_/F. The elimination rate constant from the central compartment is k_e_. The transfer rate constant from the central compartment to the superficial compartment is denoted as k_12_ and the transfer rate constant from the superficial compartment to the central compartment as k_21_. α (alpha) and β (beta) are exponents. A and B are coefficients in the sum of exponentials. Apparent total clearance of the drug from plasma after oral administration corrected with the value of bioavailability is denoted as Cl/F.

Formulas used to calculate pharmacokinetic parameters for the one-compartment model with lag time are as follows:

$$K_{el}=\alpha \;$$

$$V_{c}=\frac{Dose}{A}$$

$$AUC=A\frac{\;K_{a}}{K_{a}-\alpha }\left(\frac{1}{\alpha }-\frac{1}{K_{a}}\right)$$

$$AUMC=\left[A\frac{K_{a}}{K_{a}-\alpha }\left(\frac{1}{\alpha^{2}}-\frac{1}{K_{a}^{2}}\right)\right]$$

$$MRT=\frac{AUMC}{AUC}-\left(lag+\frac{1}{K_{a}}\right)$$

$$T_{1/2\alpha }=\frac{\text{ln}2}{\alpha }$$

$$Cl=\frac{Dose}{AUC}$$

$$V_{ss}=Cl\cdot MRT$$

$$V_{Z}=\frac{Cl}{\lambda z}$$

The formulas used to calculate pharmacokinetic parameters for the extravascular fitted two-compartment model are as follows:

$$a=K_{el}+K_{12}+K_{21}$$

$$b=a^{2}-4K_{el}\cdot K_{21}$$

$$\alpha =\frac{1}{2}\left(a+\sqrt{b}\right)$$

β=a−α

$$A=C_{o}\left(\frac{K_{21}-\alpha }{\beta -\alpha }\right)$$

$$B=C_{o}-A$$

$$V_{c}=\frac{Dose}{A+B}$$

$$AUC=\left[A\frac{K_{a}}{K_{a}-\alpha }\left(\frac{1}{\alpha }-\frac{1}{K_{a}}\right)\right]+\left[B\frac{K_{a}}{K_{a}-\beta }\left(\frac{1}{\beta }-\frac{1}{K_{a}}\right)\right]$$

Individual plasma profiles from the volunteers were analyzed, and pharmacokinetic parameters were calculated using the computer program of Thermo Kinetica software version 5.0 (Thermo Fisher Scientific Inc., United States).

### Genotyping Methods

DNA was extracted from the buccal swab samples using the QIAamp DNA Blood Mini Kit (QIAGEN, Hilden, Germany) according to the manufacturer’s instructions.

Genotyping of *CYP3A4* (−392 G>A, rs2740574), *CYP3A5* (6986A>G, rs776746), *CYP2C9* (430 C>T, rs1799853, and 1075A>T, rs1057910), and *CYP2C19* (681 G>A, rs4244285 and −806 C>T, rs12248560) was performed by TaqMan^®^ Drug Metabolism Genotyping Assays (Thermo Fisher Scientific, USA) on ViiA Real-Time PCR System (Applied Biosystems™, Foster City, CA, USA) according to manufacturer’s protocol.

*CYP1A1* (1759A>G, rs4646903) genotypes were analyzed by polymerase chain reaction-restriction fragment length polymorphism (PCR-RFLP), using the MspI restriction enzyme (New England Biolabs, Hitchin, United Kingdom), as previously described (Feldman et al., 2009).

### Statistical Analysis

All statistical analyses were carried out using SPSS version 26.0 software (IBM, USA, 2019). The continuous variables were presented as the median [with interquartile range (IQR): 25th and 75th percentiles] or geometric mean with standard deviation and categorical variables as number (percent). For continuous variables, comparisons between groups (gender) were made using the Mann–Whitney test for non-normally distributed data. The Kolmogorov–Smirnov test was used to confirm the nonparametric distribution of parameters. Categorical data were compared using the Chi-squared test. Multiple linear regression analysis has been performed using data (AUC_∞_) obtained from non-compartmental analysis of itraconazole and hydroxy-itraconazole as dependent (target) variables with age, gender, and body mass index as independent variables. Linear regression analysis was performed for AUC, AUC_corr_, C_maxcalc_, and C_maxcalc corr_ between one-compartmental and two-compartmental models. It was shown as goodness-of-fit plots, whereas the association between these parameters (one-compartmental *vs*. two-compartmental model) has been performed by Pearson’s correlation. The value of *p* < 0.05 was considered significant throughout the analyses. The association between genotypes and genders was analyzed by two-tailed Fisher exact test.

## Results

Demographic data, physical examinations, and laboratory tests of the study participants are shown in Table 1. All findings were within the reference values in accordance with the study protocol.

**TABLE 1:** Demographic, clinical, and laboratory characteristics of participants.

Evaluated characteristics of participants	*n* = 38
Age	37.00 (33.75–41.00)
Body weight (kg)	77.50 (68.75–90.00)
Body height (cm)	177.50 (171.75–183.00)
BMI (kg/m^2^)	25.22 (22.99–27.41)
RBCs (x 10^12^/L)	4.76 (3.67–5.01)
Hemoglobin (g/L)	135.50 (126.00–145.25)
Hematocrit	0.42 (0.39–0.45)
WBCs (x 10^9^/L)	6.60 (5.71–7.51)
Platelets (x 10^9^/L)	247.00 (220.00–273.50)
Glucose (mmol/L)	5.00 (4.70–5.30)
Creatinine (mmol/L)	66.00 (52.75–76.50)
AST (IJ/L)	21.00 (19.75–23.25)
ALT (IJ/L)	22.00 (17.75–29.50)
ALP (U/L)	132.00 (115.50–159.00)
Proteins (g/L)	72.00 (69.75–74.00)
Albumin (g/L)	44.00 (43.00–45.00)
Uric acid (µmol/L)	297.00 (243.00–354.25)
Blood urea nitrogen (mmol/L)	4.90 (4.28–5.73)
Total cholesterol (mmol/L)	4.34 (4.08–4.56)
Total bilirubin (µmol/L)	9.50 (7.00–12.25)
Triglycerides (mmol/L)	0.92 (0.64–1.23)

Data are presented as the median [with interquartile range (IQR): 25th and 75th percentiles]. BMI, body mass index; RBCs, red blood cells; WBCs, white blood cells; AST, aspartate aminotransferase; ALT, alanine transaminase; ALP, alkaline phosphatase.

Non-compartmental analysis of pharmacokinetic parameter AUC∞ of itraconazole for all human subjects showed that its geometric mean (geomeans) ± standard deviation (SD) value was 673.23 ± 491.40, while the corresponding value for hydroxy-itraconazole was 908.48 ± 753.11. Statistically significant models are obtained by multiple linear regression analysis if AUC_∞_ of itraconazole and hydroxy-itraconazole, as well as the sum of itraconazole and hydroxy-itraconazole, is a dependent variable, whereas age, gender, and body mass index are independent variables. It was shown by three tested models that gender is the only significant variable associated with the largest percentage of the variability of all independent variables (Table 2).

**TABLE 2:** Multiple linear regression analysis for AUC_∞_ of itraconazole (ITR) and hydroxy-itraconazole (HITR), as well as the sum of ITR and HITR obtained from non-compartmental analysis as a dependent (target) variable with age, gender, and body mass index as independent variables.

	Coefficient	Standard error	*p*-value
AUC_∞_ of itraconazole^a^			
Gender	−211.349	100.823	0.038
Age	6.883	7.055	0.331
BMI	−3.514	18.691	0.851
AUC∞ of hydroxy-itraconazole^b^			
Gender	−412.737	148.411	0.006
Age	−3.797	10.385	0.715
BMI	41.109	27.513	0.138
Sum of AUC_∞_ of itraconazole and hydroxy-itraconazole^c^			
Gender	−624.086	211.970	0.004
Age	3.086	14.832	0.836
BMI	37.595	39.296	0.341

a F = 2.075; *p* = 0.108; R = 0.231 (23.1%).

b F = 5.330; *p* = 0.002; R = 0.356 (35.6%).

c F = 4.902; *p* = 0.003; R = 0.343 (32.3%).

**FIGURE 1:**
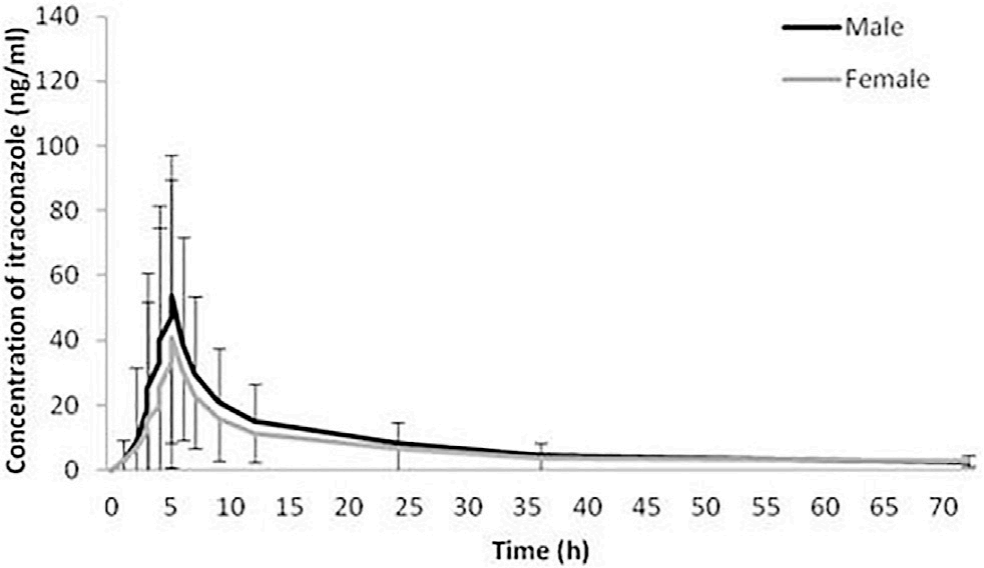
Plasma concentration-time curves. Geometric mean and standard deviation of itraconazole plasma concentrations over time after a single oral dose of 100 mg of itraconazole in 114 analyzed sets of plasma concentrations, 66 obtained from male and 48 from female healthy Caucasian subjects.

**FIGURE 2:**
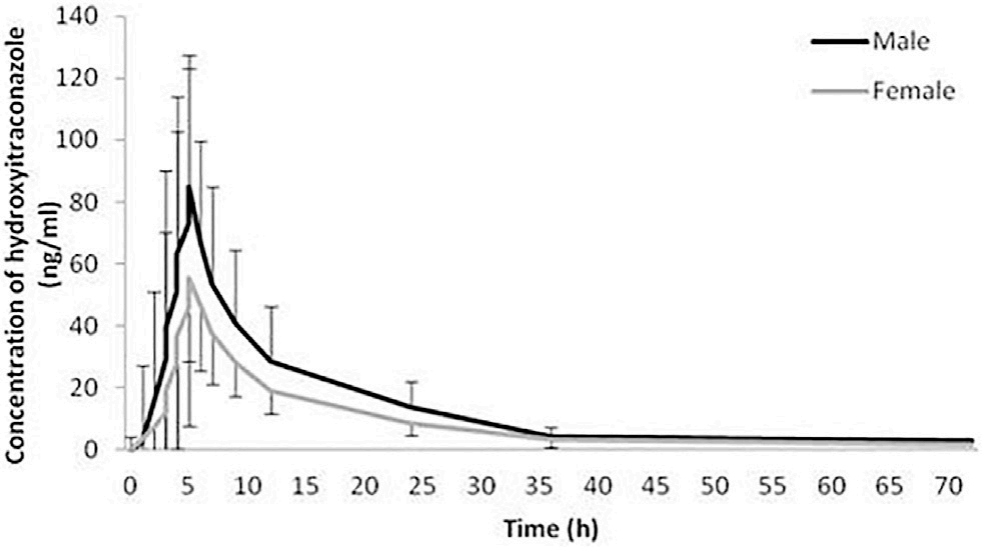
Plasma concentration-time curves. Geometric mean and standard deviation of hydroxy-itraconazole plasma concentrations over time after a single oral dose of 100 mg of itraconazole in 114 analyzed sets of plasma concentrations, 66 obtained from male and 48 from female healthy Caucasian subjects.

The geomean±SD plasma concentration-time curves of itraconazole and hydroxy-itraconazole obtained after a single dose of 100 mg of itraconazole oral administration in males and females are shown in Figures 1, 2. Obtained concentrations were used to perform pharmacokinetic analyses.

Statistical analysis has shown that geomean of itraconazole concentrations in men were higher than those in women, reaching statistical significance from the third to the 12th hour after administration of itraconazole. Similarly, geomean of hydroxy-itraconazole concentrations in men was higher than in women during the whole examined period of 72 h. Moreover, this difference was statistically significant from the second to the twenty-fourth hour after itraconazole application.

**FIGURE 3:**
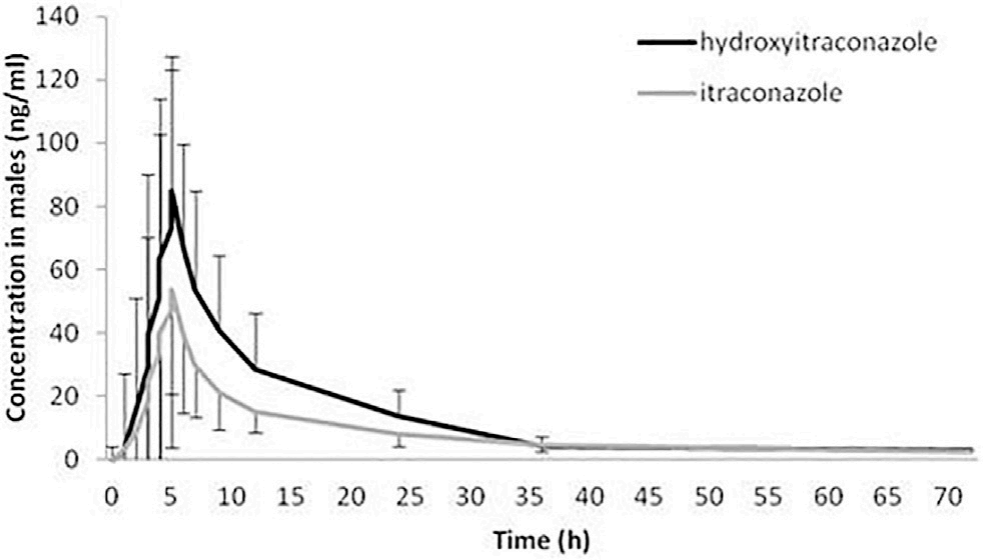
Plasma concentration-time curves of itraconazole and hydroxy-itraconazole after a single oral dose of 100 mg of itraconazole providing 66 sets of plasma concentrations from male healthy Caucasian subjects. Data are presented as geometric mean and standard deviation.

**FIGURE 4:**
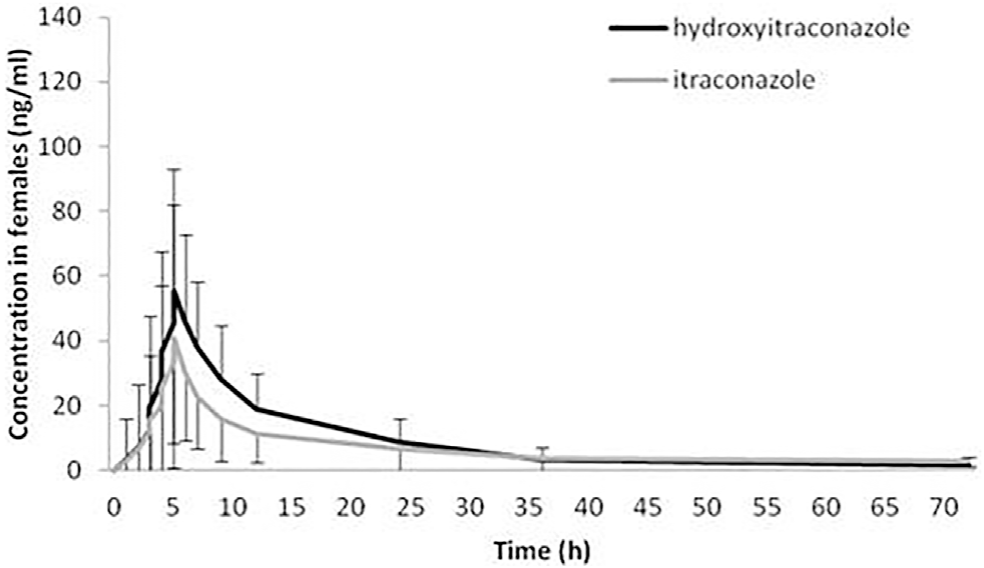
Plasma concentration-time curves of itraconazole and hydroxy-itraconazole after a single oral dose of 100 mg of itraconazole providing 48 sets of plasma concentrations from female healthy Caucasian subjects. Data are presented as geometric mean and standard deviation.

The geomean hydroxy-itraconazole concentrations were higher than the geomean itraconazole concentrations in all subjects, reaching statistical significance during the whole examined period of 72 h. In men, significance was achieved from the first to the twenty-fourth hour after administration of itraconazole, while in women, a significant difference between drug and metabolite concentrations, in favor of metabolite, was achieved from the fourth to the twenty-fourth hour after application of itraconazole (Figures 3, 4).

### Non-Compartmental Analysis

The geomean of pharmacokinetic parameters determined by non-compartmental analysis is summarized in Tables 3, 4. Analysis of pharmacokinetic parameters of itraconazole showed that women had significantly lower median values of C_max_ and AUC_72h_ in comparison to men (Table 3). This statistical difference was even more noticeable when the values of these parameters were corrected according to the body weight of the subjects (i.e. when C_maxcorr_ and AUC_72hcorr_ were considered). In contrast, the difference between genders concerning parameter AUC_∞_ became significant after correction by the body weight. Namely, AUC_∞ corr_ for men is significantly higher than for women (632.46 ± 495.19 *vs*. 401.46 ± 249.36, respectively). Pharmacokinetic parameter t_max_ of itraconazole was not significantly different between genders (Table 3). The geomean of V_d_/F was significantly higher in females than in males. Values of Cl/F of itraconazole were similar in both genders (Table 3). The same applies to k_e_ and t_1/2_ of itraconazole.

**TABLE 3:** Pharmacokinetic parameters of itraconazole calculated from its 114 sets of plasma concentrations in both genders of human subjects after a single oral dose of 100 mg of drug obtained by non-compartmental analysis.

Variable itraconazole plasma samples	Men *n* = 66	Women *n* = 48	*p*-value
C_max_ (ng/ml)	59.82 ± 48.49	44.65 ± 35.02	0.016
C_maxcorr_ (ng/mL/mg/kg)	50.41 ± 44.92	30.92 ± 24.52	<0.001
t_max_ (h)	5.29 ± 0.54	5.42 ± 0.36	0.648
AUC_72h_ ((h)*(ng/ml))	623.75 ± 442.87	473.95 ± 356.96	0.022
AUC_72h corr,_ ((h)*(ng/ml)/mg/kg)	525.62 ± 400.70	328.19 ± 230.33	<0.001
AUC_∞_ (h)*(ng/ml)	750.55 ± 540.65	579.75 ± 387.28	0.061
AUC_∞ corr,_ ((h)*(ng/ml)/mg/kg)	632.46 ± 495.19	401.46 ± 249.36	<0.001
V_d_/F (L/kg)	44.48 ± 47.25	69.42 ± 55.43	0.002
t_1/2_ (h)	20.61 ± 27.54	19.27 ± 25.91	0.872
Cl/F (L/h)	133.24 ± 86.56	172.49 ± 124.54	0.061
k_e_ (h^−1^)	0.03 ± 0.03	0.04 ± 0.08	0.872

*C*_*max*_, peak concentration; *t*_*max*_, time to reach peak concentration; *AUC*_*72h*_, area under the concentration-time curve from time 0 to the time of the last sample with the quantifiable concentration (*C*_*72h*_); *AUC*_*∞*_, area under the concentration-time curve from time 0 extrapolated to infinity; *C*_*maxcorr*_, *AUC*_*72hcorr*_, *and AUC*_*∞*__*corr*_, corresponding parameters corrected according to the body weight of the subjects; *V*_*d*_ */F*, apparent volume of distribution after non-intravenous administration; *Cl/F*, apparent total clearance of the drug from plasma after oral administration; *k*_*e*_, terminal elimination rate constant; *t*_*1/2*_, terminal half-life (additional explanations are given in the text). Values are presented as geometric mean and standard deviation.

**TABLE 4:** Pharmacokinetic parameters of hydroxy-itraconazole calculated from the 114 sets of plasma concentrations obtained from both genders of human subjects after application of a single oral dose of 100 mg of itraconazole using non-compartmental analysis.

Variables hydroxyitraconazole	Men *n* = 66	Women *n* = 48	*p*-value
C_max_ (ng/ml)	93.39 ± 57.16	62.48 ± 36.54	<0.0001
C_maxcorr_ (ng/mL/mg/kg)	78.69 ± 53.74	43.27 ± 26.09	<0.0001
t_max_ (h)	5.36 ± 0.46	5.54 ± 0.54	0.316
AUC_72h_ ((h)*(ng/ml))	985.75 ± 781.20	633.60 ± 319.85	<0.0001
AUC_72h corr_ ((h)*(ng/ml)/mg/kg)	830.66 ± 758.47	438.75 ± 224.44	<0.0001
AUC_∞_ ((h)*(ng/ml))	1,090.40 ± 885.56	706.84 ± 360.82	<0.0001
AUC_∞ corr_ ((h)*(ng/ml)/mg/kg)	918.85 ± 850.37	489.47 ± 258.32	<0.0001
t_1/2_ (h)	12.16 ± 11.39	11.96 ± 11.54	0.927
k_e_ (h^−1^)	0.06 ± 0.05	0.06 ± 0.08	0.927

*C*_*max*_, peak concentration; *t*_*max*_, time to reach peak concentration; *AUC*_*72h*_, area under the concentration-time curve from time 0 to the time of the last sample with the quantifiable concentration (*C*_*72h*_), *AUC*_*∞*_, area under the concentration-time curve from time 0 extrapolated to infinity; *C*_*maxcorr*_, *AUC*_*72hcorr*_, *AUC*_*∞*__*corr*_, corresponding parameters corrected according to the body weight of the subjects; *k*_*e*_, the terminal elimination rate constant; *t*_*1/2*_, terminal half-life (additional explanations are given in the text). Values are presented as geometric mean and standard deviation.

Pharmacokinetic parameters of hydroxy-itraconazole, the main metabolite of itraconazole administered *per os*, as a single 100 mg capsule, are summarized in Table 4. As obtained for itraconazole, significantly fewer geomean values of C_maxcorr_, AUC_72hcorr_, and AUC_∞ corr_ were found in women compared to men. Pharmacokinetic parameter T_max_ of hydroxy-itraconazole was not significantly different between genders. The same applies to the median values of k_e_ and t_1/2_ of hydroxy-itraconazole.

### Compartmental Analysis

The compartmental analysis was also carried out using the individual human subject’s plasma profile of itraconazole concentrations. The application of the pharmacokinetic one-compartment open model was possible in 113 sets of plasma concentrations of itraconazole out of 114. By calculating pharmacokinetic parameters, we obtained statistically significant gender differences in the following parameters: C_maxcalc_, C_maxcalc corr_, t_maxcalc_, AUC_corr_, and V_d_/F (Table 5). The geomean values of corrected C_maxcalc_ and AUC were significantly lower in females than males. On the contrary, geomean values of t_maxcalc_ and V_d_/F were significantly higher in women than in men. k_e_ and Cl/F values were similar in both genders.

**TABLE 5:** Pharmacokinetic parameters of itraconazole calculated from sets of plasma concentrations of human subjects after administration of a single oral dose of 100 mg of drug obtained by one-compartment model analysis.

Variable one-compartment model analysis	Men *n* = 66	Women *n* = 47	*p*-value
k_a_ (h^−1^)	0.87 ± 5.55	0.67 ± 0.67	0.256
t_lag_ (h)	2.42 ± 0.97	1.35 ± 0.89	0.496
t_max calc_ (h)	4.85 ± 0.96	5.25 ± 0.97	0.021
C_maxcalc_ (ng/ml)	48.39 ± 40.64	33.60 ± 29.70	0.006
C_maxcalc corr_ (ng/mL/mg/kg)	40.78 ± 37.46	23.25 ± 20.32	<0.001
AUC ((h)*(ng/ml))	380.68 ± 292.54	331.62 ± 349.78	0.314
AUC_corr_ ((h)*(ng/ml)/mg/kg)	380.68 ± 292.54	229.44 ± 225.09	0.009
Vd/F (L/kg)	14.18 ± 21.42	24.04 ± 23.18	0.002
k_e_ (h^−1^)	0.22 ± 0.16	0.18 ± 0.19	0.435
α	0.22 ± 0.16	0.18 ± 0.19	0.435
Cl/F (L/h)	262.69 ± 229.82	301.55 ± 316.42	0.314

*k*_*a*_, absorption rate constant; *t*_*lag*_. lag time; *C*_*maxcalc*_, calculated maximum concentration; time where t = C_max_-t_max calc_; *Vd/F*, apparent volume of distribution of the central compartment after oral administration in a one-compartment model; *AUC*, area under the plasma concentration-time curve; *C*_*maxcalc corr*_, *AUC*_*corr*_, corresponding parameters corrected according to the body weight of the subjects; *k*_*e*_, elimination rate constant from the central compartment; *α*, exponent; *Cl/F*, apparent total clearance of the drug from plasma after oral administration. Values are presented as geometric mean and standard deviation.

**FIGURE 5:**
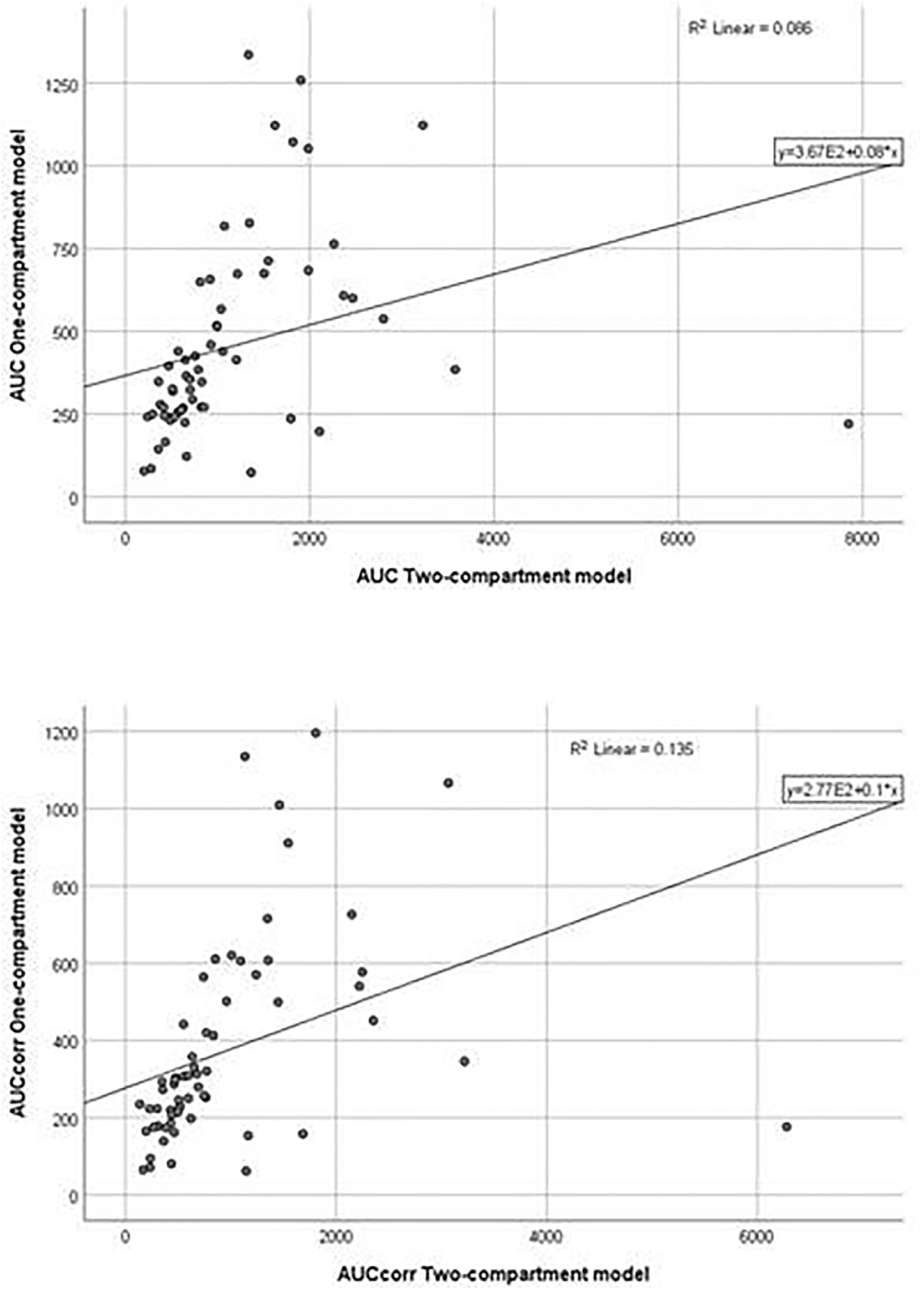
Goodness-of-fit plots for the one-compartment model *versus* the two-compartment model (AUC, AUC_corr_).

**FIGURE 6:**
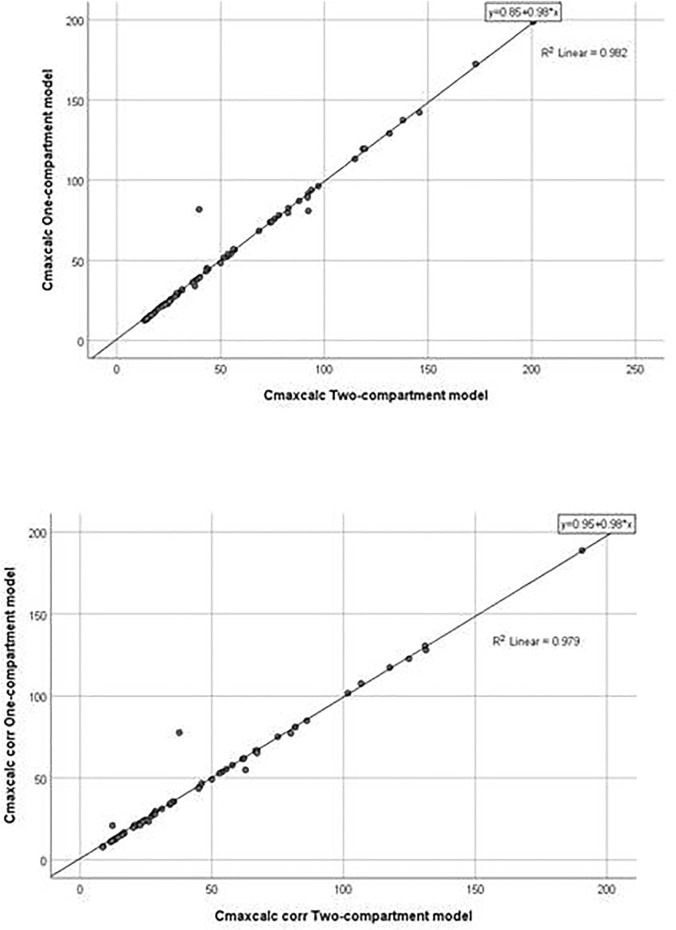
Goodness-of-fit plots for the one-compartment model *versus* the two-compartment model (C_maxcalc_ and C_maxcalc corr_).

The application of the pharmacokinetic two-compartment open model was possible in 64 sets of plasma concentrations of itraconazole out of 114. By processing pharmacokinetic data, a significant difference in the geomean of corrected parameter C_maxcalc_ was found. In other words, it was significantly lower in women than in men. However, the geomean of the parameter V_z_/F of itraconazole was significantly higher in women than in men. All other calculated parameters did not differ between genders, except for coefficient in the sum of exponentials B, which was significantly lower in females. Results are not shown, but linear regression analysis between the selected pharmacokinetic parameters obtained by one-compartmental and two compartmental models has been performed and presented in Figures 5, 6. This analysis has shown a positive strong correlation between one-compartment model and two-compartment model for parameters AUC and AUC_corr_ (r = 0.294; *p* = 0.021 and r = 0.368; *p* = 0.03, respectively), as well as for C_max_ and C_maxcoor_ (r = 0.991, *p* < 0.001 and r = 0.990; *p* < 0.001, respectively). On the contrary, there was no correlation between the one-compartment and two-compartment models for parameter V_d_/F.

### Genotyping Analysis

**FIGURE 7:**
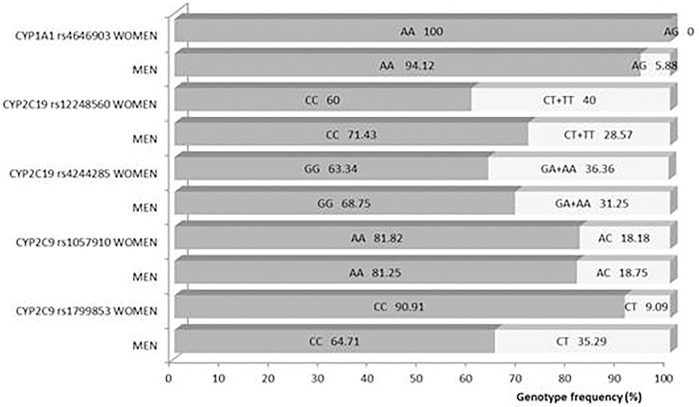
Difference of genotype frequencies between genders.

Obtained frequencies of genotypes met the Hardy–Weinberg equilibrium for all analyzed loci except for monomorphic CYP3A4 392 and CYP3A5 6986 (all participants had the same allele). Frequencies of analyzed genotypes were not significantly different between genders (Figure 7).

## Discussion

Itraconazole, a well-known triazole antifungal agent, has highly variable pharmacokinetics due to inconsistent absorption, complex metabolism, saturable elimination, and numerous interactions with concomitant medications (Hardin et al., 1988; Fagiolino et al., 2007; Allegra et al., 2017; Lindsay et al., 2017). As a result, it is HVD, meaning that its within-subject variability for C_max_ and AUC is larger than 30% (European Medicines Agency, 2010; Dragojevic-Simic et al., 2018; Endrenyi and Tothfalusi, 2019). Additional sources of its pharmacokinetics variability are various and not yet fully examined, such as its different pharmaceutical formulations, age, body mass index, ethnicity, and aforementioned gender of the patient (Fagiolino et al., 2007; Allegra et al., 2017). When multiple linear regression analysis was performed using AUC_∞_ obtained from non-compartmental analysis of itraconazole and hydroxy-itraconazole as a dependent variable and age, gender, and body mass index as independent variables, gender was the only significant variable associated with the largest percentage of variability of all independent variables. There are conflicting results concerning the effect of age on the itraconazole pharmacokinetic parameters even in the population ranging from infants to adolescents (Abdel-Rahman et al., 2007; Shi and Klotz, 2011; Bury et al., 2021). Therefore, it is not surprising that we did not show this effect in our study, concerning that healthy subjects from 23 to 55 years old were included. In contrast, the body weight was identified as an important covariate for the population pharmacokinetics of itraconazole in children (Henning et al., 2006; Abdel-Rahman et al., 2007) and healthy adults subjects (Abuhelwa et al., 2015). Abuhelwa et al. showed that weight affected itraconazole pharmacokinetics, but gender did not (Abuhelwa et al., 2015). However, in our model, this was not the case, and it is important to stress that the tested pharmacokinetic parameter (AUC∞) of both itraconazole and hydroxy-itraconazole, which was included in the multiple linear regression analysis, was not corrected according to the body weight of the subjects as previously explained (the PK value was divided by the ratio of the given dose of the drug (100 mg) and the body weight expressed in kilograms). Namely, this analysis convincingly showed, using three tested models, that gender is the only significant variable associated with the largest percentage of the variability of all independent variables. Some other authors (Fagiolino et al., 2007; Allegra et al., 2017) also showed that the gender of the subjects has a significant influence on itraconazole pharmacokinetics. Therefore, we continued our research to prove it through detailed pharmacokinetics and pharmacogenomic analyses.

In our study, both geomean values of itraconazole and hydroxy-itraconazole concentrations were higher in men than in women, reaching statistical significance from the third to the twelfth hour and from the second to the twenty-fourth hour after itraconazole application, respectively. Moreover, the geomean hydroxy-itraconazole concentrations were higher than the geomean itraconazole concentrations in all included subjects during the whole examined period. In men, significance was achieved from the first to the twenty-fourth hour, whereas in women, the same findings were present from the fourth to the twenty-fourth hour after the application of itraconazole. Similar to other studies performed on healthy volunteers, absorption of the itraconazole from the capsules, especially after a full meal, was rather slow, with peak concentrations reached within 1.5–5 h (Hardin et al., 1988; Poirier and Cheymol, 1998; Lestner and Hope, 2013). As far as hydroxy-itraconazole is concerned, being the major circulating metabolite in human plasma (Heykants et al., 1982; Barone et al., 1993), its plasma concentrations are much higher than the parent drug (Poirier and Cheymol, 1998; Lestner and Hope, 2013), and that was the case in our investigation. Relevant pharmacokinetic parameters obtained using the standard non-compartmental and compartmental methods were calculated due to the concentration-time data for itraconazole and hydroxy-itraconazole in plasma. Both non-compartmental and compartmental analyses in our study indicated significant gender differences in itraconazole pharmacokinetics after administration of the single oral dose of the drug, given as 100 mg capsule, immediately after a full meal. Gender differences in response to treatment have been reported for various drugs as a result of physiological differences and differences in their pharmacokinetics and pharmacodynamics (Pleim et al., 2003; Soldin and Mattison, 2009; Zucker and Prendergast, 2020). Much more is known about the differences in pharmacokinetics, and changes related to it may guide alterations in dosage regimen and/or the need for TDM to increase its effectiveness or reduce potential adverse drug reactions. In some previous studies, individual pharmacokinetic values were obtained from non-compartmental (Yun et al., 2006; Abdel-Rahman et al., 2007; Dragojevic-Simic et al., 2018) and one compartmental model (Henning et al., 2006), which best fitted the data of itraconazole and hydroxy-itraconazole. In contrast, other studies reported that the pharmacokinetics of itraconazole could be well described using a two-compartmental model (Yun et al., 2006). Koks et al. tested a two-compartment model for both itraconazole and hydroxy-itraconazole in a Thai cohort of HIV-infected patients who were using itraconazole as an addition to their antiretroviral therapy (Koks et al., 2003). In the population pharmacokinetic analysis of itraconazole in pediatric patients, Bury et al. reported a three-compartment model that best fitted the data (Bury et al., 2021). There is even a more sophisticated option in which the final itraconazole model was a two-compartment model with oral absorption described by four-transit compartments, while hydroxyitraconazole was best described by a one-compartment model (Abuhelwa et al., 2015).

Non-compartmental analysis of pharmacokinetic parameters of both itraconazole and hydroxy-itraconazole indicated significantly less geomean values of C_maxcorr_, AUC_72hcorr_, and AUC_∞ corr_ in women than men. Corrections were performed according to the body weight of the subjects to exclude its influence on the calculated pharmacokinetic parameters and substantiate that gender itself is the factor that had a significant influence on them. Application of the open one-compartment model with lag time enabled us to substantiate this finding because statistically significant differences in the geomean corrected values of C_maxcalc_ and AUC in favor of the reduction in female gender were obtained. Physiological parameters that affect the drug absorption process differ to some extent in men and women, such as the differences in the level of gastric acid secretion, since some studies indicated that it was lower in women, while gastric emptying time, gastric fluid flow, and intestinal motility were higher in men (Soldin and Mattison., 2009; Soldin et al., 2011; Islam et al., 2017). All these facts are in favor of higher drug absorption in men. As itraconazole is a highly lipophilic drug, poorly soluble in water, and ionized only at low pH, its solubility in water is satisfactory only in extremely acidic conditions, such as in a gastric environment (Abulhelwa et al., 2015; Abulhelwa et al., 2016). The importance of gastrointestinal pH and transit time on itraconazole dissolution and absorption was further substantiated by the *in vitro* and *in vivo* correlation model for Sporanox and SUBA-itraconazole formulations in fed and fasting conditions (Abulhelwa et al., 2015; Abulhelwa et al., 2016). Hydrochloric acid secretion is lower in women, which would lead to less dissociation of itraconazole, its poorer absorption, and a lower rate of gastric emptying and motility. Parameters k_a_ and t_lag_ of itraconazole were not different between genders, but they are very variable, even in the same person in different conditions, and suitable mostly for designing a multiple-dose regimen (Shargel et al., 2012). Therefore, the rate of absorption in our study is reliably expressed by C_max_ and t_max_. As the geomean values of C_maxcalc_ were significantly lower in women while t_max calc_ was significantly higher in women than in men, which was obtained only using the one-compartment model, it can be concluded that the rate of absorption of itraconazole was lower in females than in males. In addition, because AUC, as a measure of total systemic exposure to the drug, was significantly lower in women in the non-compartmental and one-compartmental analysis, poorer absorption of itraconazole in women can be suggested in accordance with previous results that females have less oral drug bioavailability due to gastric pH and lower dissolution efficiency (Fagiolino et al., 2007)**.** Moreover, linear regression analysis between the selected pharmacokinetic parameters obtained by one-compartmental and two compartmental models has shown a positive, strong correlation between these two models for parameters AUC and AUC_corr_, as well as C_maxcalc_ and C_maxcalc corr_.

However, a geomean value of V_d/_F was significantly higher in women than in men when the one-compartment model was used. It is generally accepted that the distribution of a drug, in addition to its physicochemical characteristics, mainly depends on the vascular and tissue volume of distribution, and the latter is primarily influenced by the ratio of lean body mass and adipose tissue mass (Meibohm et al., 2002; Soldin et al., 2011). Since we performed a dose adjustment in relation to body weight, we excluded the influence of the body mass index, which is higher in men than in women. Moreover, multiple linear regression analysis performed using itraconazole and hydroxy-itraconazole pharmacokinetics data obtained from the non-compartmental analysis did not show the effect of body mass index on it. However, generally speaking, the body adipose tissue mass is more pronounced in women than in men and itraconazole is an extremely lipophilic drug with a very large volume of distribution (Heykants et al., 1982; Prentice and Glasmacher, 2005; McEvoy, 2016); these facts could explain a significantly higher volume of distribution of this drug in females. Similarly, by applying the pharmacokinetic two-compartment open model, the geomean of the parameter V_z_/F of itraconazole was significantly higher in women than in men. In contrast, it probably contributes to our finding of significantly lower values of C_max_ and AUC of itraconazole in women compared to men because all the pharmacokinetic processes of the drug in the body take place simultaneously (Meibohm et al., 2002).

Itraconazole is eliminated mostly by CYP3A-mediated metabolic clearance, leading to many metabolites, but with hydroxy-itraconazole as the major circulating one in human plasma, which is further metabolized with the same enzymes metabolizing itraconazole (Heykants et al., 1982; Poirier and Cheymol, 1998). In our study, in addition to the obtained lower C_max_ and AUC values for both itraconazole and hydroxy-itraconazole in females, women had, for a longer time, significantly lower geomean plasma hydroxy-itraconazole concentrations than men, compared with the duration of lower itraconazole concentrations, and the metabolite/drug concentration ratio in favor of metabolites lasted shorter in women than in men. All of this indicated that women were significantly less exposed to both hydroxy-itraconazole and itraconazole than men, but it was, to some extent, more pronounced related to the metabolite. Considering CYP P450 enzymes, since *in vitro* experiments indicated that the *CYP3A4* and *CYP1A1* subfamily catalyzed the metabolism of itraconazole to hydroxy-itraconazole, we included it in our genotyping analysis (Shimada et al., 1994; Isoherranen et al., 2004), as well as the *CYP3A5* and *CYP2C* subfamily. However, neither the difference in the frequency of the analyzed genotypes between genders nor the pharmacokinetic parameters related to elimination, such as k_e_ and Cl/F, were shown. However,, significant differences in hepatic and probably intestinal enzyme expression between genders cannot be ruled out because the *in vitro* and *in vivo* population pharmacokinetic model has shown a significant effect of hepatic extraction and hepatic blood flow on itraconazole first-pass metabolism (Abuhelwa et al., 2018). This is similar to the previous consideration by Fagiolino et al. (2007), which concluded that although k_e_ of itraconazole displayed similar values in both genders, more intense pre-systemic elimination at the enterocyte and/or hepatocyte should not be ruled out. Furthermore, this assumption would also contribute to our findings of significantly lower geomean values of C_max_ and AUC of itraconazole in women compared to men, obtained by both non-compartmental and compartmental analyses. Moreover, the non-existence of significant differences in geomean values of k_e_ and Cl/F between genders after a single dose does not mean that it is the case in real clinical situations because the elimination of itraconazole and hydroxy-itraconazole is a saturable process and the itraconazole excretion mechanism is saturated at therapeutic doses (Sweetman, 2009; McEvoy, 2016; Datapharm, 2021). The significance of these facts could be reflected in the findings that women have a 1.5–1.7-fold greater risk of developing adverse drug reactions than men because gender differences in pharmacokinetics strongly enable the prediction of gender-related adverse drug reactions, which is not the case in men (Anderson, 2005; Islam et al., 2017; Zucker and Prendergast, 2020). Therefore, equal inclusion of males and females in clinical trials is necessary, as well as the assessment of adverse drug reactions based on research and clinical evidence after the appearance of the drug at the market (Watson et al., 2019). The other very important clinical implication of our work derives from our results providing better arguments for the introduction of routine TDM of itraconazole and related triazoles in hospital settings because more rational pharmacotherapy of fungal infections in patients of both genders is needed nowadays.

### Study Limitations

The limitation of the study is reflected in the small number of subjects who were examined. However, because 114 itraconazole and hydroxy-itraconazole sets of plasma concentrations (i.e., 1,824 plasma samples of healthy subjects in total) were obtained for pharmacokinetic analyses, valid statistical calculations could be performed. However, there are not too many studies dealing with the identification of factors influencing the pharmacokinetics of itraconazole, especially differences concerning body mass index, gender, and age in humans, in order to provide better answers related to the sources of its variability.

## Conclusion

Multiple linear regression analysis indicated that gender had a significant effect on AUC as the most important pharmacokinetics endpoint, whereas body mass index and age did not show such an influence. Both non-compartmental and compartmental analysis indicated significant influence of gender on itraconazole pharmacokinetics after the administration of the single oral dose of the drug, given under fed conditions. In our study, non-compartmental and one-compartmental models complemented each other, while the application of the two-compartmental model showed a significant correlation with the analysis of one compartment. Women were less exposed to itraconazole and hydroxy-itraconazole than men due to poorer absorption of itraconazole, its more intense pre-systemic metabolism, and higher distribution of both drug and its metabolite. Analyzed genotypes and gender differences in drug pharmacokinetics could not be related.

## Data Availability

The datasets presented in this study can be found in online repositories. The names of the repository/repositories and accession number(s) can be found in the article/Supplementary Material.
