# Supplementary material for: Influence of Gender, Body Mass Index, and Age on the Pharmacokinetics of Itraconazole in Healthy Subjects: Non-Compartmental Versus Compartmental Analysis
Source: Front Pharmacol. 2022 Jun 15;13:796336. doi: 10.3389/fphar.2022.796336 (PMC9240599; doi:10.3389/fphar.2022.796336)
Supplement: Supplementary file 1 [file DataSheet1.docx]

**Supplementary materials**

Plasma concentrations of itraconazole and hydroxy itraconazole were determined using a validated liquid chromatography with mass spectrometry detection (LC-MS) assay modified based on the method of Patni et al. (Patni et al., 2010; Patni et al., 2012).

**Instrumentation and LC–MS analytical conditions**

The LC-MS method was developed using a liquid chromatograph Waters Aquity 2695 System separation module (HPLC binary pump equipped with autosamples/variable injector, degasser, Hypersil GOLD 100.0 x 2.1 mm, 3.0 μm Therrmo Scientific column with Hypersil GOLD 3.0 μm 10.0 x 2.1 mm guard column, maintained on 30^0^C) (HPLC, Alliance 2695, Waters, [Massachusetts](https://en.wikipedia.org/wiki/Massachusetts), USA) and mass spectrometer (ZQ, Quattro Micro, Waters, [Massachusetts](https://en.wikipedia.org/wiki/Massachusetts), USA). The mobile phase was consisted of mixture of acetonitrile and 5.0 mM ammonium-acetate (70:30, v/v) at the flow rate of 0.2 mL/min. The run time was 6 mins with retention times of approximately 2.8 mins for itraconazole, 2.3 mins for hydroxy itraconazole, and 2.2 mins for internal standard ketoconazole, respectively. Detection was performed on a mass spectrometer (ZQ, Quattro Micro, Waters, [Massachusetts](https://en.wikipedia.org/wiki/Massachusetts), USA) equipped with a electrospray positive ionization. Detector settings has been as follows: electrospray ionization (ESI), positive-polarity ionization; capillary voltage, 3.5 kV; source temperature, 110^0^C; desolvation temperature, 350^0^C; nitrogen desolvation flow, 400.0 L/h; nitrogen cone flow, 50.0 L/h. The multiple-reaction monitoring frag­mentation transitions were set at *m/z* 705.0 for the itraconazole, m/z 721.0 for hydroxy itraconazole, and *m/z* 237.0 for internal standard ketoconazole, respectively.

**Sample preparation**

The plasma samples were collected in the following pharmacokinetic times measured in hrs: 30 mins before and 1, 2, 3, 3.5, 4, 4.5, 5, 5.5, 6, 7, 9, 12, 24, 36 and 72 hrs after applying of drug, and stored in refrigerator at -70^0^C. Frozen plasma samples from subjects were thawed to room temperature before processing. After defrosting samples at room temperature, in 1.0 mL of plasma was added 200.0 μL of 5.0 mM ammonium-acetate and 100.0 μL of internal standard solution. Itraconazole and hydroxy itraconazole were extracted with 3.0 mL of diethyl ether for 20 mins. Plasma samples were centrifuged on 3000 rpm for 5 mins, and organic layer was separated and evaporated in the stream of air. Dried extract was reconstituted in 1.0 mL of mobile phase, and 50.0 μL was injected into the LC-MS system for analysis, at room temperature.

**Method validation**

LC-MS method for the determination of itraconazole and hydroxy itraconazole in human plasma was developed and validated according to the requirements of the European Medicines Agency (EMA) Guidance on Bioanalytical Method Validation (European Medicines Agency, 2011). The validation of the LC-MS bioanalytical method for determination of itraconazole (ITC) and hydroxy itraconazole (HITC) in human plasma included the following items as shown in Table 1.

**Linearity of calibration curves and low limit of quantification (LLOQ)**

Quantification was based on peak areas. Calibration curves were constructed using linear regression analysis. The calibration curve was linear over the range of Itraconazole and hydroxy itraconazole of 2.00 - 100.00 ng/mL with a coefficient of correlation (*r*^2^) ≥ 0.99. The typical equation of the calibration curve was *y* = 0.0047*x -* 0.0055*y*, *r^2^* = 0.9993 (itraconazole), and *y* = 0.01*x -* 0.0038*y*, *r^2^* = 0.9989 (hydroxy itraconazole), where *y* represents the peak area ratio of analyte derivative to the IS derivative, and *x* represents the plasma concentration, respectively. The LLOQ of this assay was 2.00 ng/mL.

**Precision and accuracy**

Intra- and inter-day precision and accuracy of itraconazole and hydroxyitraconazole are sum­marized in Table 1, as follows:

1) Itraconazole: The intra-day precision were 1.28% and 7.90%, respectively, and the accuracy ranged from 94.84% to 105.33% of the nominal concentra­tion. The inter-day precision were in the range from 1.20% to 7.78%, and the accuracy were 95.17% and 103.82%, respectively, of the nominal concentra­tion.

2) Hydroxy itraconazole: The intra-day precision were 0.88% and 6.11%, respectively, and the accuracy ranged from 99.73% to 108.82% of the nominal concentra­tion. The inter-day precision were in the range from 2.05% to 8.09%, and the accuracy were 90.13% and 102.77%, respectively, of the nominal concentra­tion.

Therefore, the intra- and inter-assay precision and the accuracy for the determination of itraconazole and hydroxy itraconazole were acceptable.

A dilution test was also performed to evaluate the preci­sion and accuracy of the method for diluted samples. Keeping in mind that some real samples may have analytes levels exceeding the maximum concentrations of the calibration curves, a past-dilution method 1 + 1 with blank plasma was validated. The dilution accuracy (calculated as CV of the replicated QC sample) have been +15% (85 - 115%), like in undiluted QC sample. Blank plasma samples (n = 4) were spiked with both analytes at 150.00 ng/mL, 110.00 ng/mL and 50.00 ng/mL of itraconazole and of hydroxy itraconazole, respectively. Before preparation, samples were diluted 1 + 1 with other blank plasma: finally the diluted QC samples were extracted and analysed. The concentrations of itraconazole and hydroxy itraconazole were then back-calculated using normal calibration curves. The estimated concentrations have been compared with the theoretical-ones, and shown in Table 2.

**Specificity of the bioanalytical method and matrix effect**

The chromatograms of blank, spiked and drug-administrated plasma samples were compared to determine the level of interference by endogenous co-eluent components in the quantification of of itraconazole and hydroxy itraconazole. Analyses were performed on 6 blank plasma samples from different healthy volunteers (including a lipemic and a haemolytic sample) without addition of internal standard then with addition of the internal standard or itraconazole or hydroxy itraconazole; no peak, interfering with those of itraconazole, hydroxy itraconazole or the internal standard, must appear. The selectivity at LLOQ concentration level is part of the limit of quantification test as outlined in a previous paragraph. No significant matrix effect was observed for analyte in the plasma samples. The possibility of matrix effect caused by ionization competition between the analytes and the endogenous co-eluents was evaluated by QC low (2.00 ng/mL) and QC high (75.00 ng/mL) samples in six replicates. The results of matrix effect were acquired from comparing the peak responses of the post extraction spiked samples with those of the standard solution and suggested negligible matrix effect in this method (Table 1).

For all the tested concentrations, the ratios of the peak response from 93.00 to 103.00% were within an acceptable range. These results indicated that ion suppression or enhancement from the plasma matrix was negligible under the current conditions.

**Criteria for quality control of quantitative data**

In each sequence of analytical runs the following parameters were verified in order to accept the analytical data:

1) Calibration graph with a coefficient of determination *r^2^* > 0.99;

2) At least 75% of these standards must be within +15% of their nominal value (+20% for the lower limit calibrator;

3) Calibration points outside these limits can be removed, except LLOQ and the highest point, provided that at least 75% of the points will remain and that the regression model doesn’t change;

4) At least two-thirds of the QC samples periodically analysed in the whole sequence, at each concentration, must be within +15% of their nominal value.

The following procedures were to be followed in case of abnormal results:

1) If a sequence has a calibration graph outside the indicated limit, all the sequence is repeated: only the results of the repetition are considered;

2) If a sequence has a correct calibration graph but the QC samples at any concentration overcome the accuracy limits, all sequence is repeated; only the results of the repetition are considered;

3) If samples present estimated concentration exceeding the upper limit of the calibration curve analyses are repeated once after past dilution with blank plasma; only the results of the diluted repetition of the analyte exceeding the limits (multiplied by the dilution factor) are considered;

4) Analyses giving concentrations below the lower limit of the calibration curve are considered equal to zero;

5) Samples not showing the peak of the internal standard due to wrong preparation and/or documented technical fault (i.e. autosampler failure, low gas pressure, partially clogged mass spectrometer orifice) are repeated once and only the results of the repetitions are considered;

6) Samples with defective labeling or not properly handled during the transport (broken tubes, open tubes, others) are repeated 3 times from the other set of samples and only the means of the repetition results are considered;

7) If samples give concentrations that do not fit adequately in the individual pharmacokinetic profile, as evaluated by the pharmacokinetic responsible, or the chromatography is not satisfactory the same samples are repeated 3 times; the mean values of these replicates and of all measurements for the critical analyte are reported in this report and are used for the final pharmacokinetic calculations. In case of time 0 samples if the individual values of the 3 repetitions are below LLOQ the original value is not considered in calculation as for wrong prepared samples.

**Stability**

The plasma samples were stored at -70^0^C. Spiked plasma samples were stored in these conditions up to 2 weeks in order to exclude significant degradation of itraconazole and hydroxy itraconazole. An extended stability (up to 6 months) was validated next, when the prepared spiked samples were accomplished this storage period. The stability was also tested on spiked plasma samples at room temperature up to 6 hrs. These tests were carried out to evaluate the itraconazole and hydroxy itraconazole stability during the sample preparation and extraction. The stability of spiked samples was finally evaluated after 3 freeze-thawing cycles in order to verify if samples can be re-analyzed, if needed, after repeated freezing without compromising the results. The stability comparison must be ±15% in comparation to the control. Stability was evaluated at concentration levels QC low and QC high for itraconazole and hydroxy itraconazole, and results are sowed in Table 1.

# References

1. Patni, A.K., Monif, T., Khuroo, A.H., et al. (2010). A comparative bioavailability study of two formulations of itraconazole 100 mg capsule in healthy human Indian subjects under fasting conditions. Clinic Res Regul Aff. 27:128–132.
2. Patni, A.K., Monif, T., Khuroo, A.H., et al. (2012). Determination of pharmacokinetics of itraconazole in healthy Indian subjects under fed condition and incurred sample analysis using a validated liquid chromatography tandem mass spectrometric method. Clinic Res Regul Aff. 29:35–40.
3. European Medicines Agency (EMA). (2011). Committee for Medicinal Prod­ucts for Human Use (CHMP). Guideline on Bioanalytical Method Validation. EMEA/CHMP/EWP/192217/2009, 21 July 2011.

**Table 1** The validation of the LC-MS bioanalytical method for determination of itraconazole (ITC) and hydroxy itraconazole (HITC) in human plasma.

| **Parameter** | | **RSD (%)** | | **Ratio (%)** | |
| --- | --- | --- | --- | --- | --- |
|  |  | **ITC** | **HITC** | **ITC** | **HITC** |
| Matrix | Human plasma | - | - | **-** | - |
| Assay Volume Required | 50.00 µL | - | - | **-** | - |
| Analytical Method | LC-MS | - | - | **-** | - |
| Lower Limit of Quantification (LLOQ) | 2.00 ng/mL | - | - | **-** | - |
| Calibration curve range | 2.00 - 100.00 ng/mL | - | - | **-** | - |
| Regression Type (weighting) | Linear (1/X) | - | - | **-** | - |
| Quantitation Method | Area response | - | - | **-** | - |
| Intra-day Precision and Accuracy  (LLOQ/  QC low/  QC medium_1_/  QC medium_2_/  QC high) | Conforms | 1.28/  6.87/  7.90/  4.42/  1.73 | 6.11/  3.16/  4.71/  2.50/  0.88 | 105.33/  94.84/  96.19/  100.91/  100.9 | 104.46/  99.83/  108.82/  102.72/  99.73 |
| Inter-day Precision and Accuracy  (LLOQ/  QC low/  QC medium_1_/  QC medium_2_/  QC high) | Conforms | 2.89/  7.78/  5.79/  3.45/  1.20 | 8.09/  2.36/  7.22/  2.46/  2.05 | 103.82/  95.31/  95.17/  98.35/  99.13 | 102.72/  99.71/  90.13/  98.24/  100.21 |
| Specificity | Conforms | - | - | - | - |
| Stock stability (on-bench) | 5 hrs | 5.13/2.18 | 4.17/1.87 | 99.73 | 97.03 |
| Plasma stability (on-bench)  (QC low/QC high) | 4 hrs | 9.85/2.76 | 5.92/3.74 | 92.67/97.71 | 101.91/103.9 |
| Plasma stability -70^0^C  (QC low/QC high) | 90 days | 5.79/3.30 | 7.44/4.42 | 92.18/98.97 | 98.40/103.14 |
| Freeze-thaw  (QC low/QC high) | three cycles | 7.10/3.45 | 6.88/4.18 | 92.19/99.47 | 98.93/102.36 |
| Stability on the instrument  (QC low/QC high) | 12 hrs | 4.11/1.81 | 8.13/2.87 | 97.27/99.70 | 90.22/98.99 |
| Carry-over | With carry-over | - | - | - | - |
| Matrix factor (LLOQ/QC high) | 6 lots | 6.23/2.40 | 5.41/3.71 | 95.84/95.30 | 96.79/97.94 |
| Recovery | Conforms | - | - | 68.83 - 84.28 | 73.01 - 99.34 |

**Table 2** The concentrations (C) of itraconazole (ITC) and hydroxy itraconazole (HITC) in human plasma.

| **C before dilution (ng/mL)** | | **C after**  **dilution**  **(ng/mL)** | | **C after calculation (ng/mL)** | | **C after calculation**  **(%)** | | **Coefficient of variation**  **(%)** | |
| --- | --- | --- | --- | --- | --- | --- | --- | --- | --- |
| **ITC** | **HTC** | **ITC** | **HTC** | **ITC** | **HTC** | **ITC** | **HTC** | **ITC** | **HTC** |
| 150.00 | 150.00 | 75.00 | 75.00 | 70.79 | 69.27 | 94.39 | 95.39 | 3.34 | 2.74 |
| 110.00 | 110.00 | 55.00 | 55.00 | 52.26 | 49.14 | 95.01 | 87.05 | 4.29 | 2.06 |
| 50.00 | 50.00 | 25.00 | 25.00 | 24.19 | 22.66 | 96.75 | 88.52 | 5.68 | 5.20 |
